# Substitution of Goat’s Milk for Sucking-Bottle

**Published:** 1873-12

**Authors:** 


Substitution of Goat's Milk from the Living Animal for the Sucking Bottle.—Dr. H. MacCormac maintains that goat's milk may be abstracted from the living animal and transferred at once to the infant's stomach by means of a tube provided with an artificial nipple. In this way, it is thought, the mortality hitherto attendant upon artificial lactation may be greatly diminished. *Boston Medical Journal, Oct.* 23, 1873.

